# Long-Term Patterns of Bone Mineral Density in an Elite Soccer Player

**DOI:** 10.3389/fphys.2021.631543

**Published:** 2021-02-25

**Authors:** Chiara Milanese, Valentina Cavedon, Giuliano Corradini, Aiace Rusciano, Carlo Zancanaro

**Affiliations:** ^1^Laboratory of Anthropometry and Body Composition, Department of Neurosciences, Biomedicine and Movement Sciences, University of Verona, Verona, Italy; ^2^A.C. ChievoVerona, Verona, Italy

**Keywords:** DXA, bone, competitive season, football, least significant change

## Abstract

Changes in bone mineral density (BMD) have been associated with association football (soccer) participation. Seasonal changes in BMD of soccer players have been proposed as well. However, previous investigations were based on short-term observations. Actually, longitudinal investigation of BMD in soccer players is lacking, possibly because of frequent inter-club transfer, changes in club policy or continued availability of the relevant facilities. Dual-energy X-ray Absorptiometry (DXA)-measured areal BMD (aBMD) was obtained during the competitive season in an elite soccer player along 10 consecutive years. Findings showed that (1) aBMD tends to increase with age, independently of body mass; (2) The right (preferred, kicking) leg has higher aBMD than the left (non-preferred, support) one; (3) Meaningful (i.e., >least significant change, LSC) changes in aBMD take place along the season; and (4) The off-season (transition) period has no effect on aBMD. Findings prompt for future research aimed at clarifying the long-term and seasonal patterns of bone characteristics in soccer in relation with age and type/dose of training. Season-around, long-term scrutiny of bone status in soccer players would help controlling for possible changes/asymmetries in bone mineralization/strength.

## Introduction

Bone mineral density (BMD; i.e., the amount of mineral per unit bone volume) is a widely used, standardized parameter in the evaluation of bone strength, representing a key variable when assessing bone quality. It has been demonstrated that reducing bone mineral content or density is associated with an increase in fracture risk (Kopperdahl et al., 2014; Curtis et al., 2016). Nutrition is an important modifiable factor that affects bone health. A beneficial impact on BMD has been shown with higher adherence to a “healthy” dietary pattern like the Mediterranean diet (Movassagh and Vatanparast, 2017) and, to a moderate extent, with higher protein intake (Shams-White et al., 2017). Also, physical exercise is associated with improved BMD in both the general and athletic population (Lupsa and Insogna, 2015; Maillane-Vanegas et al., 2020). BMD is affected by the individual’s hormonal status as well (e.g., parathyroid hormone, thyroid hormones, growth hormone, and estrogens; Grabowski, 2015). While true BMD (g/cm^3^) can be measured using quantitative CT or MRI (Di Iorgi et al., 2018), the widespread use of these techniques is hampered by high radiation dose and/or cost; accordingly, in this work, we used Dual-energy X-ray Absorptiometry (DXA), an accurate, low-radiation, widespread method to measure areal BMD (aBMD, g/cm^2^; Jain and Vokes, 2017). aBMD contributes 70% of mechanical strength at the femur and 40% at the spine (Austin et al., 2012) and is clinically feasible, the assessment of bone tissue and mechanical properties being limited to a few research facilities specialized in skeletal biomechanics.

Association football (hereinafter, soccer) is considered an odd impact loading sport placing dynamic, multidirectional loads on the body with a potential for inducing positive skeletal adaptations in both young and elderly people (Helge et al., 2014; Zouch et al., 2015). aBMD was found to be higher in soccer players than in elite long-distance runners (a different impact-loading sport) at specific sites suggesting sport-specific effect of soccer practice on the skeleton (Fredericson et al., 2007). In lifelong-trained male soccer players, aBMD of the proximal femur and whole-body BMD were markedly higher at age 65–80 years compared to age-matched untrained men (Hagman et al., 2018). Interestingly, lower limb skeletal asymmetries were found in young male soccer players (12–18 years), possibly as a result of performing side specific movements such as shooting, passing, and jumping. In male professional soccer players, soccer participation was found to be associated with increased aBMD vs. age and body mass index-matched subjects (Wittich et al., 1998; Suarez-Arrones et al., 2019). However, all of these studies where cross-sectional in design, thereby preventing assessment of the long-term, longitudinal patterns of aBMD in soccer players. Such an assessment would be relevant to sport science and sports medicine, because BMD changes can have substantial implications for athlete health and injury risk in the elite sporting environment (Herbert et al., 2019). In professional soccer, fractures represent a limited number of total injuries (Ekstrand et al., 2011). However, most fractures were classified as major injuries (Eirale et al., 2013).

In professional soccer leagues, a typical season lasts about 11 months, inclusive of the pre-season period (from early July to mid-August) the competitive season proper (from mid-August to end of May) and an off-season (transition) period of 4–6 weeks. aBMD in athletes is likely to change during the course of the competitive season and/or sports career as a result of e.g., training and competition (Karlsson et al., 1996; Stanforth et al., 2016). Seasonal changes are expected to take place in soccer players as well, who may be training at different loads along the season. However, the seasonal patterns of BMD in soccer are not well defined. Statistically significant, non-consistent changes in BMD were found along the competitive season in both adult male (Milanese et al., 2015) and female (Minett et al., 2017) players as well as young male players (Suarez-Arrones et al., 2019), but their clinical value was limited due to the short time span of follow-up.

It is apparent that long-term aBMD data collection, possibly at different season periods, would improve knowledge on bone characteristics changes associated with soccer participation. Study of this kind is difficult in soccer players essentially because of frequent inter-club transfer, changes in club policy or continued availability of the relevant facilities. The present paper reports on the results of a retrospective study carried out in a male adult elite soccer player where DXA was used to analyze aBMD over an extended period of time with an aim at getting insight into the long-term and seasonal pattern of aBMD.

## Materials and Methods

This was a retrospective study with data coming from routine testing, which was part of formal program of sport science support. The study protocol conformed to the Helsinki declaration (revised 2008), and was approved by the proper IRB at the local University (#26/2019). Informed, written consent was obtained from the participant.

The formal program of sport science support consisted in three whole-body DXA scans per year. Accordingly, measurements took place at three season time-points: early July (at the beginning of the conditioning period: pre-season), January/February: mid-season, and May (at the end of the competitive season: end-season). The minimum interval between two consecutive scans was 9 weeks. Comparing aBMD at the end of the competitive season with that at the beginning of the next one allowed insight at aBMD changes during the off-season or transition period, which is considered to elicit negative effect on body composition (Silva et al., 2016) in association with reduced training.

### Participant Characteristics

The player was a forward playing in the top Italian soccer championship (Serie A) for the 10 consecutive competitive seasons considered in this work. At the beginning of data collection, he was 30-year-old, his body mass was 75.9 kg, and his stature was 174.1 cm. In the period considered in this study, the player played a total of 16,486 min in 263 Serie A matches scoring 62 goals. Minutes played per season ranged 453 (2017/2018)–3,093 (2010/2011). One major (>2 months) ligaments injury (ankle) was registered at the end of the 2012/2013 competitive season; in the other seasons, injury duration was 7,9,8,5,5,9,12,27, and 43 days with longest stops in January/March of the 2016/2017, 2017/2018, and 2018/2019 seasons. No inter-club transfer took place in the period investigated in this study. The player was reportedly non-smoker and never suffered from bone fracture. No long-term corticosteroid treatment or chronic disease was found in history. The player’s dietary habits conformed to the Club’s advice (Mediterranean diet) and did not change to any important extent in the observation period. Across the season, the player had typically six training sessions a week with an average duration of 70 min. Training was comprised of standard macrocycle sand microcycles typical of soccer and involved a balanced mix of aerobic power work and soccer-specific technical exercises and gestures. The balance was shifted in favor of the latter component in late February. All over the season, players performed isotonic, isometric, and eccentric exercises twice a week.

### DXA Body Composition Analysis

A QDR Explorer W scanner (Hologic, MA, United States; software for Windows XP version 12.6.1) was used. Over the 2007–2019 period the device showed negligible baseline drift (0.991 ± 0.041 g/cm^2^, slope of the regression line = −0.058/y). In our laboratory, the short-term test-retest error for aBMD is 0.9% (with repositioning). All scanning and analysis were performed by the same skilled technician. Participant DXA positioning and scanning were in accordance with the manufacturer’s guidelines. Care was taken to avoid overlap of body regions, particularly between arms and trunk. Regions of interest were analyzed manually using the default regions determined by the Hologic software. For the purpose of this study, aBMD values were also calculated for the right and left arm combined (Arms). Evaluation of more clinically relevant regions (e.g., femoral neck or Ward’s triangle) could not be carried out because these measurements were not included in the formal program of sport science support. On each DXA measurement, the participant body mass was taken at the nearest 0.1 kg with an electronic scale (Tanita BWB-800 MA). Stature was measured with a Harpenden stadiometer (Holtain Ltd., Crymych, Pembs, United Kingdom) at the nearest mm.

### Statistical Analysis

Normality of data distribution was assessed with the Shapiro-Wilk test. In case of non-normal distribution, data were log transformed. Means were compared using repeated-measure ANOVA or the Student’s *t*-test as needed. For *t*-test, the effect size *d* was calculated as per Cohen (1988) and rated small (0.2), medium (0.5), or large (0.8). For ANOVA, homogeneity of variance was assessed with the Levene test and sphericity with the Mauchly test; effect size was calculated as partial eta squared (*ƞ*_p_^2^) and evaluated according to Cohen (1988) as small (0.01), medium (0.06), and large (0.14). The strength of the correlation coefficient (Pearson’s r) was rated as per Hopkins (2009). Changes in aBMD at the three season time points were evaluated through the application of Least Significant Changes (LSC). LSC were calculated from our precision data using three repeated DXA measurements (with repositioning) of 15 kinesiology students according to published guidelines (Hangartner et al., 2013). LSC derived precision error was calculated as root mean square SD (RMS-SD), with LSC subsequently derived as RMS-SD × 2.77 (95% CI). Changes at each season time point were plotted and visually interpreted using Bland-Altman analysis. Such a plotting allows for immediate visualization of the frequency and sign (increase/decrease) of meaningful changes in body composition (Lees et al., 2017). Changes > LSC are interpreted as “true” changes (Hangartner et al., 2013). The regression coefficient B was used to estimate yearly change in aBMD using age as the independent variable. Statistical significance was set at *p* ≤ 0.05. Data are summarized as mean ± SD. The IBM-SPSS statistical package (v.25, IBM Corp., Armonk, NY, United States) was used for analysis. Decimal age was calculated with the YEARFRACT function in Excel (Microsoft Corporation, v.16.24).

## Results

**Figure 1:**
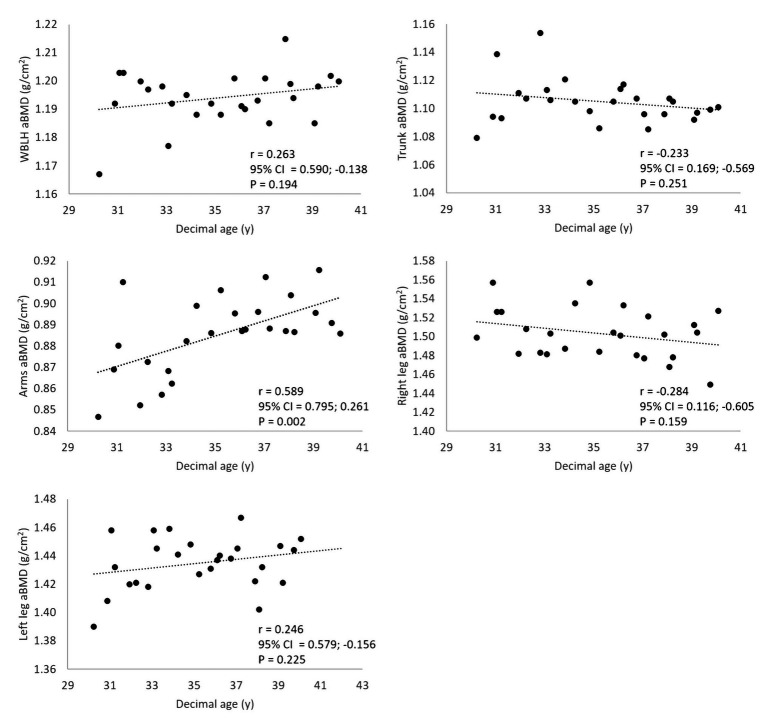
Scatterplots of aBMD at several skeletal sites plotted against age. 95% CI = 95% upper and lower CIs.

Measurement was taken at 26 time points from 2009 to 2019. Scale-measured body mass and DXA-measured total mass were highly correlated (*r* = 0.989, *p* < 0.001). In this period, the participant’s Z score ranged 2–2.7 and T score 1.7–2.3. No statistically significant correlation was found between age and body bone area, (*r* = 0.140, *p* = 0.494) nor between aBMD and body mass (*r* = 0.104, *p* = 0.615), fat-free soft tissue mass (*r* = 0.112, *p* = 0.355), and % body fat (*r* = −0.033, *p* = 0.872). Figure 1 shows scatterplots of aBMD at several skeletal sites plotted against age. aBMD showed a small, non-statistically significant decrease with age at the trunk and right leg site, and a small, non-statistically significant tendency to increase with age at the whole-body less head (WBLH) and the left leg site. A large, statistically significant increase with age was found at the Arms site (*r* = 0.589, *p* = 0.002).

Areal BMD showed statistically significant higher values in the right vs. left (non-preferred, support) leg (right leg, 1.503 ± 0.027; left leg, 1.435 ± 0.019 g/cm^2^; *t* = −11.452, *p* < 0.001, *d* = 2.913). Regression analysis estimated a statistically significant yearly increase for Arms (adjusted *R*^2^ = 0.269; *p* = 0.004), and left leg (adjusted *R*^2^ = 0.298; *p* = 0.002) aBMD. The corresponding equations were as follows: Arms aBMD, 0.726 + (0.005 × age), SE of the estimate = 0.021 g/cm^2^; left leg aBMD, 1.310 + (0.004 × age), SE of the estimate = 0.016 g/cm^2^.

Repeated-measure ANOVA showed no statistically significant main effect of season measurement time point on aBMD (Table 1), albeit a large effect size was present for WBLH, Arms, and Trunk aBMD. During off-season, no statistically significant aBMD change was found (*t*-test, Table 2), effect size being small to medium.

**Table 1:** Seasonal changes in bone mineral density (BMD; g/cm^2^) at different body sites. Repeated-measure ANOVA. Data were from the six seasons for which complete (pre-season, mid-season, and end-season) information was available.

Variable	Pre-season	Mid-season	End-season	*F*	*p*-value	Eta squared	Power
WBLH	1.187 ± 0.011	1.200 ± 0.008	1.195 ± 0.010	2.525	0.113	0.252	0.427
Arms	0.886 ± 0.024	0.897 ± 0.041	0.889 ± 0.016	0.180	0.841	0.083	0.064
Left leg	1.434 ± 0.029	1.443 ± 0.013	1.429 ± 0.018	4.686	0.089	0.701	0.445
Right leg	1.497 ± 0.017	1.505 ± 0.027	1.499 ± 0.022	0.184	0.839	0.084	0.064
Trunk	1.095 ± 0.014	1.109 ± 0.023	1.111 ± 0.015	1.693	0.293	0.458	0.193

WBLH, whole-body less head.

**Table 2:** Changes in areal BMD (aBMD; g/cm^2^) at different body sites during off-season. Student’s *t*-test. Data were from the six seasons for which complete (pre-season, mid-season, and end-season) information was available.

Variable	Seasonal measurement time point		*t*	*p*-value	*d*
	End-season	Pre-season			
WBLH	1.192 ± 0.010	1.193 ± 0.006	1.701	0.162	0.121
Arms	0.891 ± 0.010	0.892 ± 0.019	0.060	0.958	0.023
Left leg	1.428 ± 0.017	1.437 ± 0.021	1.089	0.322	0.412
Right leg	1.486 ± 0.025	1.506 ± 0.030	1.416	0.216	0.684
Trunk	1.110 ± 0.017	1.100 ± 0.011	−1.277	0.258	0.339

WBLH, whole-body less head.

**Figure 2:**
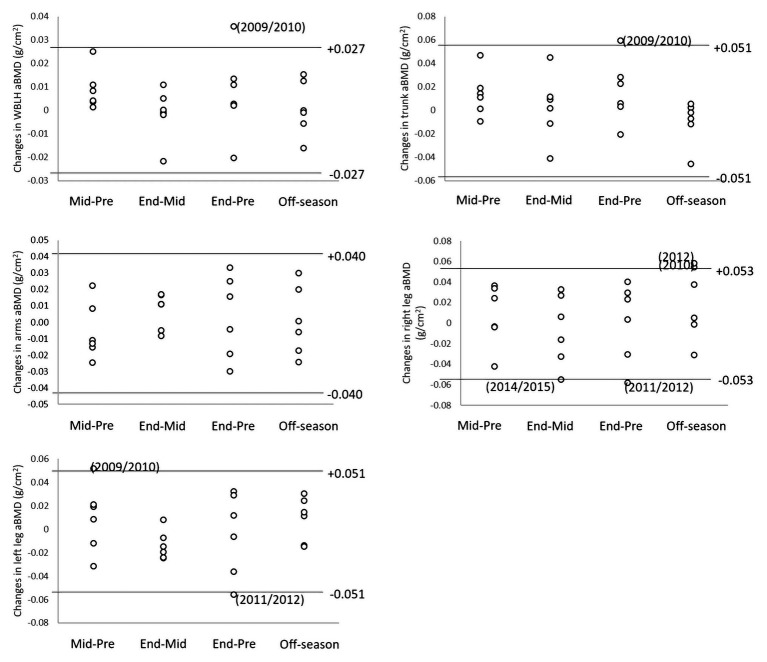
Seasonal changes in aBMD at the WBLH and regional level by least significant changes (LSC) as determined in our laboratory.

Figure 2 shows seasonal changes in aBMD at the WBLH and regional level by LSC as determined in our laboratory. Several meaningful (i.e., >LSC) changes were found at the WBLH and regional level showing that true changes in aBMD took place.

## Discussion

No age-associated changes in bone area were found in the observation period, indicating that this variable did not affect aBMD results. WBLH aBMD positively albeit non-significantly correlated with age (Figure 1). The correlation remained positive after controlling for body mass (*r* = 0.256), suggesting that the tendency of aBMD to increase with age was independent of the age-associated increase in body mass. Since data were collected at chronological age 30–39 years and considering that peak bone mass is usually achieved around 35 year of age, these findings suggest that soccer practice is able to improve bone quality up to the end of the fourth decade of life. This is supported by previous cross-sectional findings showing better aBMD in lifelong trained male soccer player vs. age-matched untrained controls (Hagman et al., 2018). Improving aBMD beyond the age at which peak bone mass is achieved in the general population may have a positive effect on the long-term bone health of the player, given the association between aBMD and injury (e.g., bone fractures, Nordström et al., 2005). Such an effect is possibly related to the high peak strains imposed on bone by soccer practice. These are applied in different directions during jumping and landing, starting and stopping, as well as transverse and torsional loads brought about by fast changes in body displacement direction (Robling et al., 2002). In soccer, applied strains may be higher than in other impact training sports (e.g., running) and therefore may stimulate higher bone mineral acquisition and better structural properties (Fredericson et al., 2007).

The right, preferred leg had a significantly higher aBMD (+4.7%) vs. the left, non-preferred one. This is at odds with previous findings in soccer players showing higher tibial bone strength values in the support leg by peripheral quantitative CT (Anliker et al., 2013) or no asymmetry in the entire leg. However, these studies were conducted in adolescent (12–18 year, Anliker et al., 2013 or young mean age 19.6 ± 1.5 year, DeLang et al., 2017) players where the concomitance of biological growth/maturation and soccer practice could confound findings. Results presented in this paper suggest that further research should be carried out on lower limbs bone characteristics in adult players.

Regression analysis yielded a quantitative estimation of age-related changes in aBMD at the Arms and non-preferred leg site. Age explained an important amount of variance (about 27 and 30% in Arms and Left leg aBMD, respectively) with a SE of the estimate as small as 2.3 and 1.1% of mean variable value, respectively. Obviously, findings cannot be generalized to the soccer player population. However, in the absence of reference data for such population, the proposed equation can be of some use for interpreting age-associated aBMD changes in soccer players.

Repeated-measure ANOVA did not show statistically significant changes in aBMD across the season indicating that, on the average, season cycling *per se* had no major effect on bone status. Nevertheless, several meaningful (i.e., >LSC) changes were found at seasonal time points in the 10-year observation period (Figure 2). Such changes represent true biological change, in absolute terms at the individual level, which exceed measurement error (Hangartner et al., 2013). A meaningful increase in WBLH aBMD was only found at the end of the first examined season (2009/2010), showing that a maximum in seasonal aBMD accrual rate was reached at the player’s youngest age in the 10-year observation period. At the regional level, aBMD meaningful changes did not show a consistent pattern apart from right leg aBMD, which decreased on two end-season time points (2011/2012, 2014/2015). These changes were not associated with major injuries in the observation period, which took place in different seasons. In soccer, player-specific changes occurring along the season are important to recognize in order to optimize training prescription and reducing injury risk the player is competing at the highest level possible (Mujika et al., 2018). Previous work on seasonal changes in DXA-measured bone variables showed that aBMD either decreases from pre- to mid-season increasing above baseline at end-season in elite soccer players (Milanese et al., 2015) or increases at end-season in young (average age, 16.1 year) male players when strength training was added to soccer participation during a whole in-season period (26 weeks; Suarez-Arrones et al., 2019). Taken together, these findings suggest that subject- or age-related heterogeneity exists in the seasonal pattern of aBMD in soccer players. The determinants of such heterogeneity warrant further investigation.

During the off-season (or transition) period, a significant reduction in sport-specific fitness may take place in soccer players in association with reduced energy expenditure (Mujika and Padilla, 2000) despite players may be involved in voluntary training or sport activity. For example, off-season has been associated with increased body-fat (Reinke et al., 2009). To the best of our knowledge, no information is available on the effect of the transition period on aBMD in soccer players. According to current data, which were obtained over several successive off-season periods, off-season was associated with a remarkable stability of aBMD, suggesting that bone quality was not affected by 5–6 week of detraining. This may be due to the short duration of the off-season period when the player was constantly engaged in everyday life’s activities at bodyweight. Recent biochemical work (Koundourakis et al., 2019) suggested that a 6-week soccer off-season detraining period negatively affected bone physiology by suppressing bone-formation rate and inducing of bone resorption. In the current study, we did not measure bone metabolism markers and were, therefore, unable to confirm the findings from Koundourakis et al. (2019). It is possible that aBMD measurement taken with a bi-dimensional technique like DXA failed to detect subtle tri-dimensional changes in bone structure. Future work using e.g., peripheral quantitative CT would help clarifying the actual impact of off-season on bone characteristics in soccer.

This study has limitations that should be mentioned. First, only one participant was evaluated. Second, while the dietary regimen suggested by the Club was constant during the observation period (Mediteranean diet), detailed information on dietary intake was not available. Therefore, changes in calcium or protein intake, which has a relationship with BMD (Tai et al., 2015; Shams-White et al., 2017) could not be analyzed. Vitamin D plasma levels, which may be related to BMD (Charoenngam et al., 2019) were not available as well. Third, information on individual training type and load was not available thereby preventing correlation between training load and the observed changes in aBMD. However, it should be kept in mind that the study was conducted on an elite athlete, where very little control measures can be applied. For example, habitual diet and lifestyle preferences could not be prescribed by the authors. Moreover, altering the daily routines of an elite athlete would have reduced the study’s ecological validity and thus reduced the applied implications of the study. The strengths of this study are the unique duration of the observation period (10 consecutive years) allowing for identification of long-term trends in aBMD, the use of and accurate and reproducible technique such as DXA for measurements, and the use of the conservative LSC criterion to assess clinically significant changes in aBMD. Taken together, the current findings are of interest for researchers involved in sports science as applied to soccer.

In summary, this unique case report spanning over 10 consecutive years shed some light on the long-term and seasonal patterns of aBMD in an elite soccer player by showing that age-associated changes take place in aBMD, independently of body mass; the right (preferred, kicking) leg consistently has higher aBMD than the left (non-preferred, support) one; the off-season period has no effect on aBMD; meaningful aBMD changes may take place along the competitive season in an elite soccer player. These findings prompt for further studies aimed at clarifying bone characteristics along the competitive career of soccer players possibly at specific, clinically relevant sites (e.g., lumbar vertebrae, hip region).

## Data Availability Statement

The raw data supporting the conclusions of this article will be made available by the authors, without undue reservation.

## Ethics Statement

The studies involving human participants were reviewed and approved by Institutional Review Board at the local University (#26/2019). The patients/participants provided their written informed consent to participate in this study.

## Author Contributions

CZ, CM, and VC: conceptualization. VC, CM, GC, AR, and CZ: data curation. CZ: writing-original draft preparation. CZ and VC: formal analysis. All authors contributed to the article and approved the submitted version.

### Conflict of Interest

GC and AR are consultants at ChievoVerona s.r.l. ChievoVerona s.r.l. had no role in decision to publish as well as paper drafting.

The remaining authors declare that the research was conducted in the absence of any commercial or financial relationships that could be construed as a potential conflict of interest.
